# Chemotherapy in the Setting of Severe Liver Dysfunction in Patients with Metastatic Colorectal Cancer

**DOI:** 10.1155/2015/420159

**Published:** 2015-05-21

**Authors:** Pashtoon Murtaza Kasi, Gita Thanarajasingam, Heidi D. Finnes, Jose C. Villasboas Bisneto, Joleen M. Hubbard, Axel Grothey

**Affiliations:** Division of Medical Oncology, Mayo Clinic, Rochester, MN 55905, USA

## Abstract

The liver is the dominant site of metastases for patients with metastatic colorectal cancer (mCRC). Depending on the timing of diagnosis and the biology of the disease, it is not uncommon for these patients to present with visceral crisis in the form of severe liver dysfunction. Treatment of these individuals is, however, difficult and challenging. The decision to consider chemotherapy in these dire circumstances entails consideration of numerous factors. If we were to focus on just the metabolism of the different drugs and biologic agents available to treat mCRC, both 5-fluorouracil and oxaliplatin alone or in combination with a monoclonal antibody are reasonable choices. Specifically, FOLFOX is a feasible and safe option in patients with mCRC with severe liver dysfunction. Choice of the biologic agent to add to the doublet chemotherapy could be individualized based on the RAS status and the clinical scenario. Based on the divergent experience of treating 2 cases and other prior reports, a summary of recommendations with a model in the form of a “therapeutic triad” is presented. The paper highlights the therapeutic challenges in patients with mCRC and severe liver dysfunction. The choice of chemotherapeutic agents and reports of other cases/series is also presented.

## 1. Background

The liver is the dominant site of metastases for patients with metastatic colorectal cancer (mCRC). Depending on the timing of diagnosis and the biology of the disease, it is not uncommon for these patients to present with visceral crisis in the form of severe liver dysfunction. The decision to consider chemotherapy in these dire circumstances is challenging and entails consideration of numerous factors. Here we present 2 cases of mCRC with severe liver dysfunction and their discordant outcomes after initiation of chemotherapy alongside a biologic agent. Based on the experience with these cases and other prior reports, a summary of recommendations with a model in the form of a “therapeutic triad” is presented.

## 2. Case Presentations

### 2.1. Case 1

**Figure 1:**
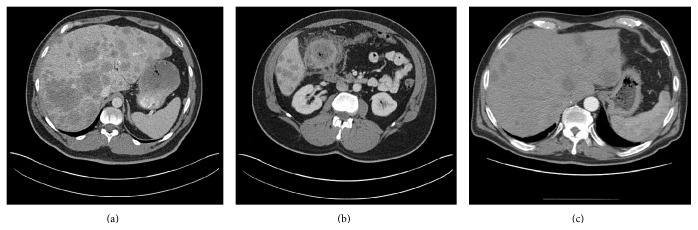
(a) CT scan of the abdomen showing innumerable liver metastases in a patient (Case 1) with colorectal cancer (a) and the primary mass in the ascending colon (b). Image (c) shows a similar scan with multiple liver metastases in another patient (Case 2) with mCRC.

Our first case is that of a 43-year-old man who presented with abdominal pain in June 2014. He developed progressive symptoms of night sweats, abdominal pain, weight loss, nausea, and vomiting over the last several weeks. On examination, he was noted to have scleral icterus, a distended abdomen, and hepatomegaly. His performance status (PS) based on the Eastern Cooperative Oncology Group (ECOG) system was 1. Computerized tomography (CT) scan revealed innumerable liver metastases (Figures 1(a) and 1(b)).

Pertinent laboratory findings are summarized in Table 1. Fine needle aspiration (FNA) cytology of a liver lesion confirmed the diagnosis of metastatic moderately differentiated adenocarcinoma. Colonoscopy confirmed the stenosing friable malignancy in the ascending colon with concern for obstruction.

While waiting for diagnostic confirmation, the patient developed fever, tachycardia, leukocytosis, and symptoms of worsening obstruction. He underwent palliative-intent right hemicolectomy. Pathology showed invasive poorly differentiated adenocarcinoma, T4a N2b (18/22) M1 Grade 3 forming a circumferential ulcerated mass in the right colon.

**Figure 2:**
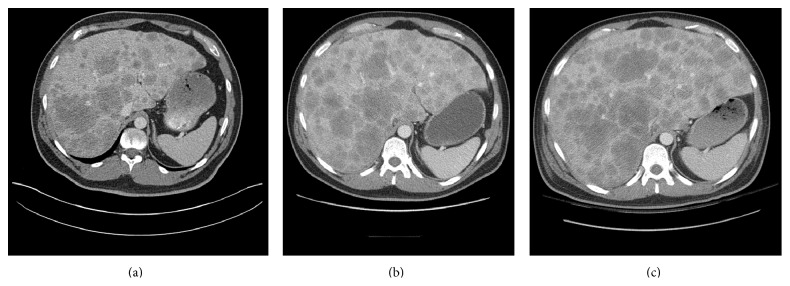
Case 1: CT scan of the abdomen showing rapid progression in both size and number of the previously noted innumerable liver metastases in a patient with mCRC: (a) day 0, (b) day 10, and (c) day 18.

One week postoperatively, he continued to deteriorate clinically and laboratory parameters are shown in Table 1. His ECOG PS now had declined to 3 from 1 earlier. Updated imaging was obtained and showed dramatic progression of disease as noted in Figure 2.

### 2.2. Case 2

Our second case is that of a 64-year-old previously healthy man who presented with fatigue and a 45-pound weight loss over three months, ECOG-1. Workup by his primary care physician was notable for iron-deficiency anemia. Colonoscopy revealed a tumor in the ascending colon. CT scan showed extensive liver metastases (Figure 1(c)). He underwent palliative-intent hemicolectomy due to bleeding from the tumor and was noted to have invasive adenocarcinoma, pT3 N2 (15/17) M1 Grade 3 with extensive synchronous liver metastases.

Two weeks later when he presented to our institution, he had developed dark urine. Exam was significant for scleral icterus and hepatomegaly. He had also declined clinically with an ECOG of 3. Pertinent laboratory findings are outlined in Table 1.

### 2.3. Therapeutic Challenges/Choice: FOLFOX ± Monoclonal Antibody

Both of our cases represented rapidly progressive mCRC with associated visceral crisis in the form of liver dysfunction from innumerable liver metastases. The decision to treat these patients with chemotherapy was complex. However, both of these patients were otherwise healthy prior to rapid clinical deterioration which was attributed in both cases to the disease. Based on consensus and understanding the risks involved, we decided to proceed with chemotherapy alongside a monoclonal antibody since in both patients the tumors were chemotherapy naïve and had not received any prior treatment apart from resection of the primary tumor.

Our first patient was started on modified FOLFOX (mFOLFOX7) with the monoclonal antibody panitumumab added later on once the tumor was noted to be RAS wild-type. Laboratory studies two days later showed significant tumor lysis in the form of hyperkalemia, hyperuricemia, and high lactate dehydrogenase (LDH) (Table 1). The patient subsequently went into renal failure with ongoing worsening liver failure and unfortunately died within 6 weeks.

Our second patient, however, did well with treatment. He was also started initially on modified FOLFOX (mFOLFOX7) with the monoclonal antibody bevacizumab added with cycle 2 of chemotherapy. Within the first cycle of treatment, the patient was noted to have improvement in his signs/symptoms (appetite/jaundice) as well as his laboratory parameters (Table 1). After 5 cycles, his laboratory findings normalized and the hepatomegaly and size of liver metastases decreased. After 10 cycles of mFOLFOX7 with bevacizumab, he was switched to FOLFIRI, followed by FOLFOX and cetuximab, and subsequently single agent cetuximab. The patient survived for 18 months from the time of diagnosis.

The following discussion relates to therapeutic challenges in patients with mCRC and severe liver dysfunction. The choice of chemotherapeutic agents and reports of other cases/series is discussed.

## 3. Discussion

The occurrence of visceral crisis in the form of liver dysfunction from liver metastases is not an uncommon phenomenon since the liver is the dominant organ of metastases for patients with mCRC. Treatment of these individuals is, however, difficult and challenging in terms of the choice and safety of the different chemotherapies and biologics [1].

These two cases represent rapidly progressive mCRC with significant deterioration within a matter of weeks. Choosing a particular chemotherapy in the setting of organ dysfunction is challenging, especially when there is ongoing decline in the patient's performance status.

If we were to focus on just the metabolism of the different drugs and biologic agents available to treat mCRC, both 5-fluorouracil and oxaliplatin alone or in combination with a monoclonal antibody are reasonable choices to consider in these patients [2–5].  Table 2 outlines the different agents available to treat mCRC in more detail. FOLFOX is a feasible and safe option in patients with mCRC with severe liver dysfunction (Table 3) [6–9]. In the palliative-intent setting, we typically avoid the 5-FU bolus since it leads to neutropenia, mucositis, and dose delays.

On the other hand, regorafenib and irinotecan would not be recommended in this subset of patients given adverse outcomes reported with respect to hepatic dysfunction (Table 2). TAS-102 is a novel antimetabolite being used for treatment of mCRC; clinical trials to evaluate its safety in patients with hepatic dysfunction are currently ongoing [10]. Mitomycin with capecitabine has also been employed in the palliative setting and does not require dose adjustments for hepatic impairment; however, the regimen is used infrequently [11].

With respect to our two cases, there was rapid deterioration of performance status in these individuals which was attributed to tumor progression. In these situations, an aggressive strategy with doublet based chemotherapy with or without a biologic should be considered. Rapidly progressive, life-threatening disease is a dire now-or-never scenario and if the decision is made to initiate treatment, it would not be appropriate to start with “chemo-light” such as 5-FU single agent.

These cases illustrate two very different extreme outcomes that can be seen in patients with mCRC and concomitant liver dysfunction. While our first patient died within weeks of initiation of chemotherapy, our second patient achieved rapid improvement of symptoms and stabilization of disease for a significant period of time after diagnosis. This is consistent with the 3 other cases reported from Roswell Park Cancer Institute [6]. Outcomes for other patients were somewhat similar with initial response followed by progression later and exposure to multiple lines of chemotherapy approved for mCRC.

**Figure 3:**
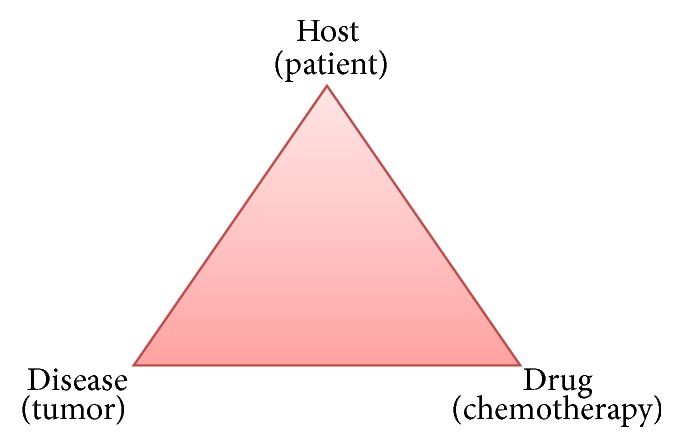
“Therapeutic triad” of the host, the drug, and the disease: factors to consider when giving chemotherapy to patients.

These cases open the debate as to if and when systemic chemotherapy with or without biologics is an option for patients with mCRC and liver dysfunction. The decision is not straightforward but can be rationalized along the lines of a “therapeutic triad” incorporating the host, the drug, and the disease, which is usually at the back of our minds when we treat any patient with chemotherapy (Figure 3).

The most important part of the triad is the host (the patient). In our cases and others described in the literature, liver dysfunction was attributed to the metastases, and the patients were otherwise healthy and active with a good overall performance status prior to the diagnosis. If these patients already had been declining and had other significant comorbidities, we would have been less inclined to consider any treatment option at all. Both of these patients had palliative-intent surgery to begin with, which signifies overall reasonable clinical status of these patients.

The second consideration is the underlying disease, in this case mCRC. Both of these tumors had no prior exposure to any chemotherapy or biologics; therefore, they were felt to be chemosensitive with potential for response and improvement in organ dysfunction [5]. This is noted in our second case and in other cases described in the literature. In patients with mCRC who develop liver dysfunction from liver metastases after multiple lines of chemotherapy, the odds of further chemotherapy leading to significant organ recovery are low. These patients may actually be harmed with more chemotherapy in the setting of liver dysfunction.

The last consideration is the choice of drug itself. As noted in Table 2, initial considerations should be made towards choosing 5-FU in combination with oxaliplatin. A monoclonal antibody could be considered initially or staggered later as noted in our cases when the RAS status is known. Even though the liver is involved in the turnover of most circulating antibodies, it is not considered rate limiting in this instance. The enzyme dihydropyrimidine dehydrogenase (DPD) metabolizes 5-FU and there are individuals with DPD deficiency that can potentially have severe and potentially lethal side effects [12]. However, genetic assessment for DPD deficiency is not routine practice but can be considered in patients who experience side effects that are out of proportion to those that would ordinarily be seen with 5-FU. With genetic tests, there is typically a long turnaround time that would limit its use in these clinical situations.

The “therapeutic triad” above in combination with other patient specific factors and goals of care can help individualize treatment decisions in patients with severe liver dysfunction and visceral crisis from mCRC.

## Figures and Tables

**Table 1:** Pertinent trends in laboratory investigations and clinical parameters for Cases  1 and  2.

	Case 1			Case 2		
	At diagnosis	Postoperative^1^	Postchemo^1^	At diagnosis^*∗*^	Postoperative^1^	Postchemo^1^
Total bilirubin	1.9	9.4	18.4	—	8.4	3.9
Direct bilirubin	1.4	8.2	16.4	—	6.2	2.4
Aspartate transaminase (AST)	679	1116	1542	—	179	82
Alanine transaminase (ALT)	314	322	—	—	—	—
Alkaline phosphatase (AlkPhos)	379	579	966	—	1708	1167
Carcinoembryonic antigen (CEA)	178.3	271.9	2770	—	0.7	—
ECOG performance status	1	3	5	1	3	0-1

^1^Postoperative and postchemotherapy laboratory investigations were roughly 1 week and 2 weeks apart, respectively.

^*∗*^At diagnosis, laboratory investigations for Case  2 were reportedly within normal limits.

**Table 2:** List of chemotherapeutic and biologic agents for patients with mCRC.

	Pharmacokinetics	Metabolism	Comments
5-Fluorouracil (5-FU) [13]	*T*_1/2_ 16 minutes (range 8–20 minutes) Excreted via lung (as CO_2_) and urine	Hepatic by dihydropyrimidine dehydrogenase (DPD) to active metabolites 5-fluoroxyuridine monophosphate (F-UMP) and 5-5-fluoro-2'-deoxyuridine-5'-O-monophosphate (F-dUMP)	Limited data in patients with total bilirubin > 5, may be treated with weekly infusion without adjustment [4].

Oxaliplatin [14]	Protein bound *T*_1/2_ 0.43 to 16.8 hours (distribution) and 391 hours (terminal) Urinary excretion	Nonenzymatic	No apparent alteration in clearance or toxicity in patients with severe liver dysfunction [3, 15, 16].

Irinotecan [17]	SN-38 protein bound *T*_1/2_ irinotecan 6–12 hours; SN-38 ~10–20 hours Excreted via urine 11–20% and via biliary tract	Hydrolyzed in the liver to active metabolite SN-38 which is further metabolized by glucuronidation by UDP-glucuronosyltransferase 1-1 (also known as UGT1A1)	In patients with varying degrees of liver dysfunction, severe side effects, poor tolerability, and overall worsening of PS were noted. [2, 18, 19]

TAS-102 [10, 20]; combination drug consisting of alpha,alpha,alpha-trifluorothymidine (FTD) and thymidine phosphorylase inhibitor (TPI)	Readily absorbed *T*_1/2_ FTD 1.4 hours, TPI 1.7–2.1 hours Limited urinary excretion FTD; TPI 29% excreted via urine	FTD undergoes hepatic metabolism, TPI minimal hepatic metabolism	Phase-I studies in patients with hepatic impairment are ongoing.

Regorafenib [21]	*T*_1/2_ for the drug 28 hours (14 to 58 hours; some metabolites may take longer for excretion, 32 to 70 hours)	Hepatic by CYP3A4 and UGT1A9	Severe drug induced liver injury has been reported with the use of drug; its use in patients with liver dysfunction would generally be avoided and requires close monitoring of liver function tests.

Monoclonal antibodies^*∗*^ [5]	*T*_1/2_ long: days to weeks	No liver metabolism/clearance	Generally acceptable to use if there are no other contraindications.

^*∗*^Anti-VEGF antibodies (bevacizumab/aflibercept) and antiepidermal growth factor (EGFR) antibodies (panitumumab/cetuximab) [1].

**Table 3:** Previous case reports of patients with mCRC and severe liver dysfunction treated with FOLFOX (reprinted with permission from JNCCN, Journal of the National Comprehensive Cancer Network [7]).

Age (y), sex	mFOLFOX6 (each drug dose: mg/m^2^)		Total bilirubin (mg/dL)		CEA (ng/mL)		Response	FOLFOX cycles until disease progression
	1st cycle progression^a^	2nd cycle^a^	Baseline	After 2 cycles	Baseline	After 2 cycles		
Case 1: 63, M [6]	Bolus 5-FU: 300, IV 5-FU: 1800, LV: 200, Ox: 60	Bolus 5-FU: 400, IV 5-FU: 240, LV: 400, Ox: 85	3.5	1.2	188.0	4.8	PR	14

Case 2: 59, F [6]	Bolus 5-FU: 0, IV 5-FU: 2000, LV: 400, Ox: 65	Bolus 5-FU: 0, IV 5-FU: 2400, LV: 400, Ox: 85	5.9	2.1	1.3	895.0	SD	8

Case 3: 37, F [6]	Bolus 5-FU: 0, IV 5-FU: 200, LV: 400, Ox: 85	Bolus 5-FU: 400, IV 5-FU: 2400, LV: 400, Ox: 85	4.2	2.1	3685.0	937.0	PR	≥10

Case 4: 67, M [7]	Bolus 5-FU: 200, IV 5-FU: 1200, LV: 200, Ox: 75	Bolus 5-FU: 200, IV 5-FU: 1200, LV: 200, Ox: 75	9.4	2.4	12.2	4.0	PR	21

Case 5: 58, F [8]	Bolus 5-FU: 0, IV 5-FU: 2400, L-LV: 200, Ox: 85	Bolus 5-FU: 400, IV 5-FU: 2400, L-LV: 200, Ox: 85	3.9	2.2	103.1	96.0	SD	6

CEA: carcinoembryonic antigen; F: female; FOLFOX: 5-FU, leucovorin, and oxaliplatin; IV: infusional; L-LV: levoleucovorin; LV: leucovorin; M: male; Ox: oxaliplatin; PR: partial response; SD: stable disease.

^a^Bolus 5-FU, infusional 5-FU, LV, and oxaliplatin.

## Abbreviations

ALT: Alanine transaminase
AST: Aspartate transaminase
AlkPhos: Alkaline phosphatase
CEA: Carcinoembryonic antigen
ECOG: Eastern Cooperative Oncology Group
KRAS: Kirsten rat sarcoma oncogene
mCRC: Metastatic colorectal cancer
PS: Performance status
VEGF: Vascular endothelial growth factor.

## References

[1] Kasi P. M., Hubbard J. M., Grothey A. (2015). Selection of biologics for patients with metastatic colorectal cancer: the role of predictive markers. *Expert Review of Gastroenterology & Hepatology*.

[2] Raymond E., Boige V., Faivre S. (2002). Dosage adjustment and pharmacokinetic profile of irinotecan in cancer patients with hepatic dysfunction. *Journal of Clinical Oncology*.

[3] Doroshow J. H., Synold T. W., Gandara D. (2003). Pharmacology of oxaliplatin in solid tumor patients with hepatic dysfunction: a preliminary report of the National Cancer Institute Organ Dysfunction Working Group. *Seminars in Oncology*.

[4] Fleming G. F., Schilsky R. L., Schumn L. P. (2003). Phase I and pharmacokinetic study of 24-hour infusion 5-fluorouracil and leucovorin in patients with organ dysfuntion. *Annals of Oncology*.

[5] Shitara K., Takahari D., Yokota T. (2010). Case Series of cetuximab monotherapy for patients with pre-treated colorectal cancer complicated with hyperbilirubinemia due to severe liver metastasis. *Japanese Journal of Clinical Oncology*.

[6] Fakih M. G. (2004). 5-Fluorouracil leucovorin and oxaliplatin (FOLFOX) in the treatment of metastatic colon cancer with severe liver dysfunction. *Oncology*.

[7] Elsoueidi R., Craig J., Mourad H., Richa E. M. (2014). Safety and efficacy of FOLFOX followed by cetuximab for metastatic colorectal cancer with severe liver dysfunction. *Journal of the National Comprehensive Cancer Network*.

[8] Shimura T., Kataoka H., Hirata Y. (2011). Metastatic colorectal cancer with severe liver dysfunction successfully treated using FOLFOX therapy. *Journal of Gastrointestinal Cancer*.

[9] Grenader T., Goldberg A., Gabizon A. (2009). Combination therapy with oxaliplatin and 5-fluorouracil in a patient with severe hepatic dysfunction associated with metastatic adenocarcinoma of the large bowel. *Anti-Cancer Drugs*.

[10] Yoshino T., Mizunuma N., Yamazaki K. (2012). TAS-102 monotherapy for pretreated metastatic colorectal cancer: a double-blind, randomised, placebo-controlled phase 2 trial. *The Lancet Oncology*.

[11] Chong G., Dickson J. L. B., Cunningham D. (2005). Capecitabine and mitomycin C as third-line therapy for patients with metastatic colorectal cancer resistant to fluorouracil and irinotecan. *British Journal of Cancer*.

[12] Caudle K. E., Thorn C. F., Klein T. E. (2013). Clinical pharmacogenetics implementation consortium guidelines for dihydropyrimidine dehydrogenase genotype and fluoropyrimidine dosing. *Clinical Pharmacology and Therapeutics*.

[13] (2007). *Adrucil [Package Insert]*.

[14] (2011). *Eloxatin [Package Insert]*.

[15] Baur M., Drescher A., Gneist M., Dittrich C., Jaehde U. (2008). Pharmacokinetics of oxaliplatin in patients with severe hepatic dysfunction. *Cancer Chemotherapy and Pharmacology*.

[16] Synold T. W., Takimoto C. H., Doroshow J. H. (2007). Dose-escalating and pharmacologic study of oxaliplatin in adult cancer patients with impaired hepatic function: a national cancer institute organ dysfunction working group study. *Clinical Cancer Research*.

[17] (2014). *Camptosar [Package Insert]*.

[18] van Groeningen C. J., van der Vijgh W. J. F., Baars J. J., Stieltjes H., Huibregtse K., Pinedo H. M. (2000). Altered pharmacokinetics and metabolism of CPT-11 in liver dysfunction: a need for guidelines. *Clinical Cancer Research*.

[19] Venook A. P., Klein C. E., Fleming G. (2003). A phase I and pharmacokinetic study of irinotecan in patients with hepatic or renal dysfunction or with prior pelvic radiation: CALGB 9863. *Annals of Oncology*.

[20] (2014). *TAS-102: Investigator's Brochure Version G 5.0 14*.

[21] (2014). *STIVARGA (Package Insert)*.

